# Endogenous testosterone density predicts unfavorable disease at final pathology in intermediate risk prostate cancer

**DOI:** 10.1007/s11255-021-02990-9

**Published:** 2021-09-27

**Authors:** Antonio Benito Porcaro, Alessandro Tafuri, Andrea Panunzio, Riccardo Rizzetto, Nelia Amigoni, Clara Cerrato, Aliasger Shakir, Sebastian Gallina, Alberto Bianchi, Francesco Cianflone, Emanuele Serafin, Alessandra Gozzo, Giacomo Di Filippo, Filippo Migliorini, Giovanni Novella, Matteo Brunelli, Maria Angela Cerruto, Alessandro Antonelli

**Affiliations:** 1 Department of Urology, University of Verona, Azienda Ospedaliera Universitaria Integrata, Piazzale Stefani 1, 37126 Verona, Italy; 2 Department of Neuroscience, Imaging and Clinical Sciences, G. D’Annunzio University, Chieti, Italy; 3 USC Institute of Urology and Catherine and Joseph Aresty Department of Urology, Keck School of Medicine, University of Southern California (USC), Los Angeles, CA USA; 4 Department of General and Hepatobiliary Surgery, Azienda Ospedaliera Universitaria Integrata, University of Verona, Verona, Italy; 5 Department of Pathology, University of Verona, Azienda Ospedaliera Universitaria Integrata, Verona, Italy; 6 Department of Urology, Vito Fazzi Hospital, Lecce, Italy

**Keywords:** Prostate cancer, Intermediate risk prostate cancer, Radical prostatectomy, Tumor upgrading, Tumor upstaging, Unfavorable disease, Endogenous testosterone, Endogenous testosterone density, Prostate-specific antigen

## Abstract

**Objective:**

To test the hypothesis that endogenous testosterone (ET) density could be associated with tumor load (TL) in patients with intermediate risk (IR) prostate cancer (PCa).

**Materials and methods:**

Endogenous testosterone density (ETD, ratio between ET and prostate volume [PV]), biopsy positive cores density (BPCD, the ratio between the number of positive cores and PV) and prostate-specific antigen density (PSAD, ratio between total PSA and PV) were retrospectively evaluated on a prospectively collected data on 430 patients with IR PCa submitted to radical prostatectomy (RP). Tumor load (TL) was measured as the percentage of prostatic volume occupied by cancer at final pathology. Unfavorable disease (UD) was defined as tumor upgrading (ISUP grading group 4, 5) and/or upstaging (pT3a or 3b) in prostate specimens. Associations were assessed by the logistic regression and linear regression models.

**Results:**

Overall, UD, which was detected in 122 out of 430 IR patients (28.4%), was predicted by BPCD (odd ratio, OR = 1.356; 95% CI 1.048–1.754; *p* = 0.020) with a sensitivity 98.4% and overall accuracy 71.9%. On multivariate analysis, BPCD was independently predicted by PSAD (regression coefficient, *b* = 1.549; 95% CI 0.936–2.162; *p* < 0.0001), ETD (*b* = 0.032; 95% CI 0.023–0.040; *p* < 0.0001) and TL (*b* = 0.009; 95% CI 0.005–0.014; *p* < 0.0001). As BPCD increased, ETD and ET levels increased accordingly, but patients with BPCD > 1.0%/mL had significantly lower ET levels.

**Conclusions:**

As ETD increased, BPCD and TL increased, accordingly; furthermore, patients with lower ET levels were more likely to have occult UD. The influence of tumor load, and unfavorable disease on ET and ETD needs to be addressed by further studies.

**Supplementary Information:**

The online version contains supplementary material available at 10.1007/s11255-021-02990-9.

## Introduction

In the aging male, prostate cancer (PCa) is the second most diagnosed tumor, which has a prevalence that increases along age groups from less than 5% by age 30 to 59% by age 80 [1]. The risk of developing PCa has been related to genetic, physical (obesity, metabolic syndrome, hypogonadism), dietary and environmental factors, as well [2]. In clinical practice, early PCa occurs frequently while investigating on associated obstructive disorders or on opportunistic screening. According to European Association of Urology (EAU) guidelines, several risk categories can be identified as related to the risk of developing metastases [2]. Among these, the intermediate risk (IR) class still represents a quite controversial issue, given that its management can largely vary from a deferred approach to multimodal active treatments [3] Noteworthily, a significant rate (from 30.3 to 46%) of IR cases are upstaged and/or upgraded at final pathology after RP, as well [4, 5]. Several authors investigated whether such events could be predicted, finding that prostate-specific antigen (PSA), PSA density (PSAD), percentage of biopsy positive cores (BPC) and tumor grade classification according to the International Society of Urologic Pathology (ISUP) are predictors of unfavorable disease (UD), which includes tumor upgrading and/or upstaging as well as high tumor load, at surgical pathology [2, 4, 5]. Accordingly, further efforts in identifying reliable predictors of UD are needed.

Endogenous testosterone (ET) is an important factor for evaluating prostate growing disorders including either benign prostatic hyperplasia (BPH) or PCa, which may also coexist [1]. Our group greatly focused its attention of the role of ET in PCa, finding that it could be associated with several unfavorable prognostic factors [6–8]. In low-risk PCa, we have recently shown that ET density (ETD), defined as the ratio of ET and prostate volume (PV), was an independent predictor of the risk of high tumor load (TL), which associated with unfavorable disease in the surgical specimen [8]. In this study, we wanted to test the hypothesis of associations of ETD with tumor load at biopsy and surgical pathology in IR disease.

## Materials and methods

### Study population

The study was approved by Institutional Review Board. Informed consent was obtained by all subjects. Data were collected prospectively but evaluated retrospectively. In a period ranging from November 2014 to December 2019, 805 consecutive PCa patients who were not under androgen blockade had ET (nmol/L) measured at our lab before surgery and the test was performed at least 1 month after biopsies between 8.00 and 8.30 a.m. by radioimmunoassay. PSA (ng/mL), age (years), body mass index (BMI; kg/m^2^), PV (mL) and percentage of BPC, the ratio of positive and total taken cores (%), were evaluated in each case. PV was calculated by TRUS standard methods. Biopsies performed elsewhere were assessed for number of cores taken, tumor grade and PV. In our institution, the 14-core trans-perineal technique was used. In each case, the ratios of BPC, PSA and ET with PV were calculated, and relative densities indicated as BPCD (%/mL), PSAD (ng/mL^2^) and ETD (nmol/(dL × mL)), respectively. Clinical staging was assessed by the 2017 version of the TNM system (8th edition) with clinical T stage only referring to DRE findings and patients were classified into risk classes, as recommended by guidelines [2].

Surgery, which was delivered by robot assisted (RARP) or open approach (ORP), was performed by experienced surgeons. Extended PLND was decided according to guidelines [2]. Nodal packets were submitted in separate packages according to a standard anatomical template including external iliac, obturator, Marcille’s common iliac, and Cloquet’s nodal stations, bilaterally [9, 10]. Removed prostates were placed into formalin, weighted and evaluated by the dedicated pathologist who graded all tumors according to the ISUP system [2]. Tumor quantitation was assessed as tumor load (TL), which was defined as percentage of prostate involved by cancer; specifically, our dedicated pathologist assessed tumor quantitation by visual estimation of all the glass slides after all microscopically identifiable foci of carcinoma have been circled with a marked pen, as considered by the ISUP association [11]. Surgical margins were stated positive when cancer invaded the inked surface of the specimen. Removed lymph nodes were assessed for number and cancer invasion. Surgical specimens were staged by the 2017 version of the TNM system (8th edition), accordingly [2].

### Statistical methods

The study wanted to test the hypothesis of associations between ETD, TL and UD features including tumor upgrading (ISUP > 3) and/or tumor upstaging (pT > 2). Continuous variables were measured for means (standard deviation, SD) and medians (interquartile range, IQR). Categorical factors were assessed for frequencies (percentages).

Associations of clinical and pathological factors with the risk of UD was evaluated by the logistic regression method (univariate and multivariate analysis); furthermore, the fit of the model was assessed by classification tables with a cut point *c* = 0.5, which is the standard for this kind of assessment; moreover, sensitivity, specificity and overall accuracy were also assessed for each model, accordingly.

Associations of clinical and pathological factors with tumor load features at either biopsy or pathology were evaluated by correlation analysis. Predictors of BPCD were evaluated by the linear regression model (univariate and multivariate analysis). Appropriate biplots were computed. The software used to run the analysis was IBM-SPSS version 26. All tests were two-sided with *p* < 0.05 considered to indicate statistical significance.

## Results

### Clinical and pathological demographics of the patient population

Overall, 430 out of 805 PCa patients (53.4%) were classified as IR class by EAU system [2]. Accordingly, ISUP grade groups were distributed as follows: grade 1 in 69 cases (16%), grade 2 in 240 subjects (55.9%) and grade 3 in 121 (28.1%) patients; clinical stage resulted cT1c in 259 patients (66.2%) and cT2 (a/b) in 171 (31.8%) cases; median (IQR) PSA was 6.4 (4.9–8.9) ng/mL. According to the preoperative physical status system, 43 patients were ASA I (10%), 351 ASA II (81.6%) and 36 ASA III (8.4%). Surgery was delivered by the robotic approach in 386 (89.8%) patients. Further details are reported in Table 1. In the surgical specimen, tumors were upgraded in 68 (15.8%) cases of whom 54 (12.6%) were ISUP grade group 4 and 14 (3.3%) grade 5. Downgrading occurred in 12 out of 240 ISUP grade 2 cases (5%) and 31 out of 121 ISUP grade 3 subjects (25.6%), respectively. Cancers were upstaged in 90 (20.9%) subjects of whom 44 (10.2%) had extracapsular extension and 46 (10.7%) seminal vesicle invasion. Tumors invaded loco regional lymph nodes in 25 out of 338 (7.4%) patients who underwent PLND. Overall, UD was detected in 122 (28.4%) cases. In the surgical specimen, median (IQR) values of TL was 19 (10–25)%.

**Table 1:** Demographics of 430 prostate cancer patients belonging to the intermediate risk class

Continuous variables	Mean (SD)	Median (IQR)
Age (years)	64.8 (6.6)	65 (61–70)
Body mass index; BMI (kg/m^2^)	25.7 (3.3)	25.6 (23.6–27.8)
Endogenous testosterone; ET (ng/dL)	434.3 (169.6)	418.9 (314.8–520.4)
ET density (ETD; ng/(dL × mL))	12.1 (8.1)	10.4 (6.9–14.4)
Prostate-specific antigen; PSA (ng/mL)	7.2 (3.5)	6.4 (4.9–8.9)
PSA density (PSAD; ng/(mL × mL))	0.19 (0.11)	0.16 (0.12–0.24)
Prostate volume (PV; mL)	42.6 (17.9)	39 (830–51)
Biopsy positive cores; BPC (%)	35.4 (20.8)	30 (20–50)
BPC density (BPCD; %/mL)	1.0 (0.8)	0.8 (0.42–1.35)
Prostate weight; PW (grams)	4.3 (18.5)	50 (41–63.5)
Tumor load (TL; %)	20.3 (13.9)	19 (10–25)

Categorical variables	Number (%)	
ISUP at biopsy		
ISUP 1	69 (16)	
ISUP 2	240 (55.9)	
ISUP 3	121 (28.1)	
Clinical T stage (cT)		
cT1c	259 (60.2)	
cT2 (a/b)	171 (31.8)	
ISUP at pathology		
ISUP < 4 (no tumor upgrading)	362 (84.2)	
ISUP > 3 (tumor upgrading)	68 (15.8)	
Pathologic tumor stage (pT)		
pT2	340 (79.1)	
pT3a	44 (10.2)	
pT3b	46 (10.7)	
Surgical margins status (SM)		
Negative	319 (74.2)	
Positive	111 (25.8)	
Pathologic nodal stage (pN)		
pN0	313 (72.8)	
pN1	25 (5.8)	
pNx	92 (21.4)	
Unfavorable disease at surgical pathology (*)	122 (28.4)	

*SD* standard deviation, *IQR* interquartile range, *ISUP* International Society of Urologic Pathology tumor grade group formulation

*Tumor upgrading and/or upstaging at surgical pathology in removed prostates

### TL associations

**Fig. 1:**
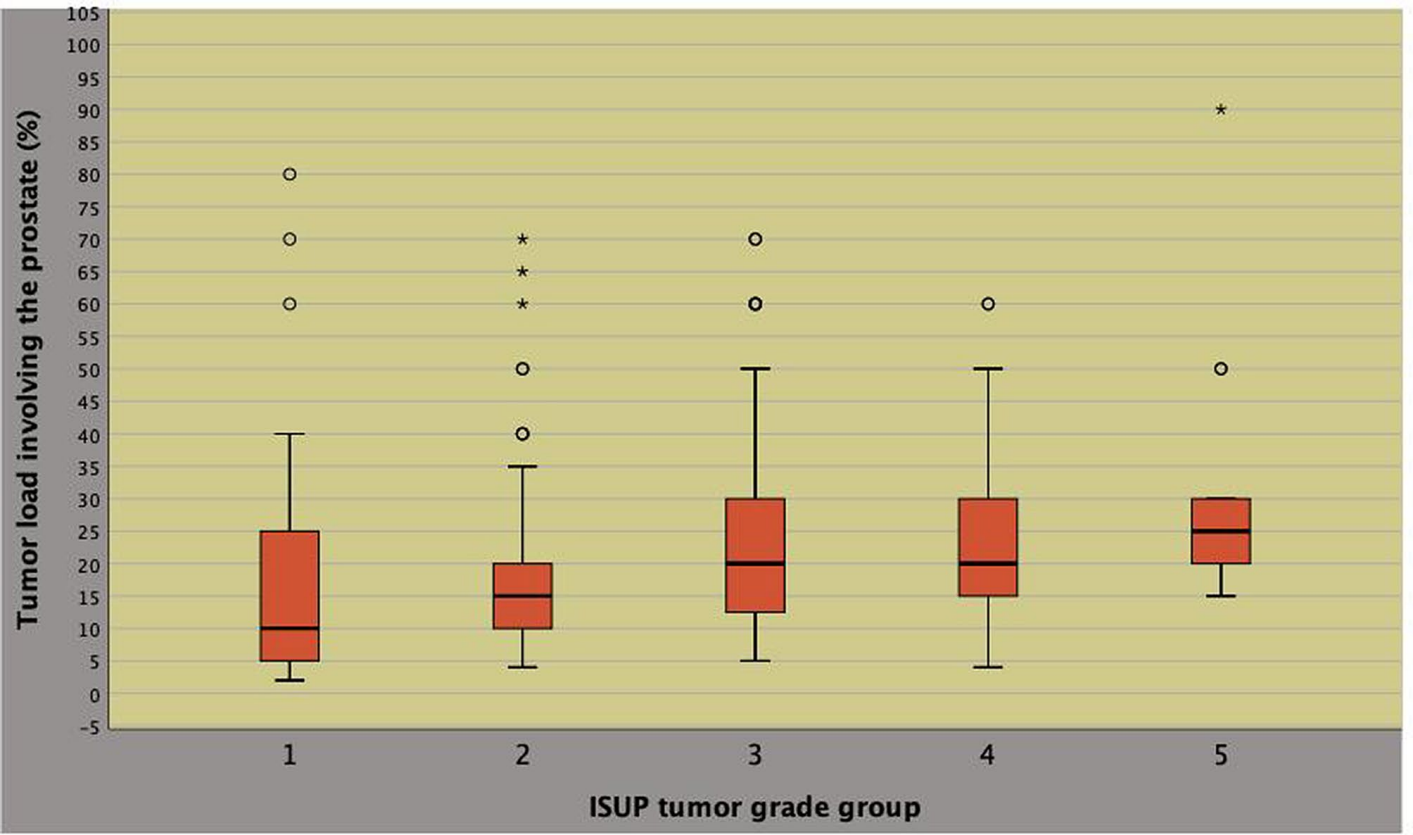
Positive correlation between tumor load and ISUP system at surgical pathology (Pearson’s correlation coefficient, *r* = 0.227; *p* < 0.0001)

**Fig. 2:**
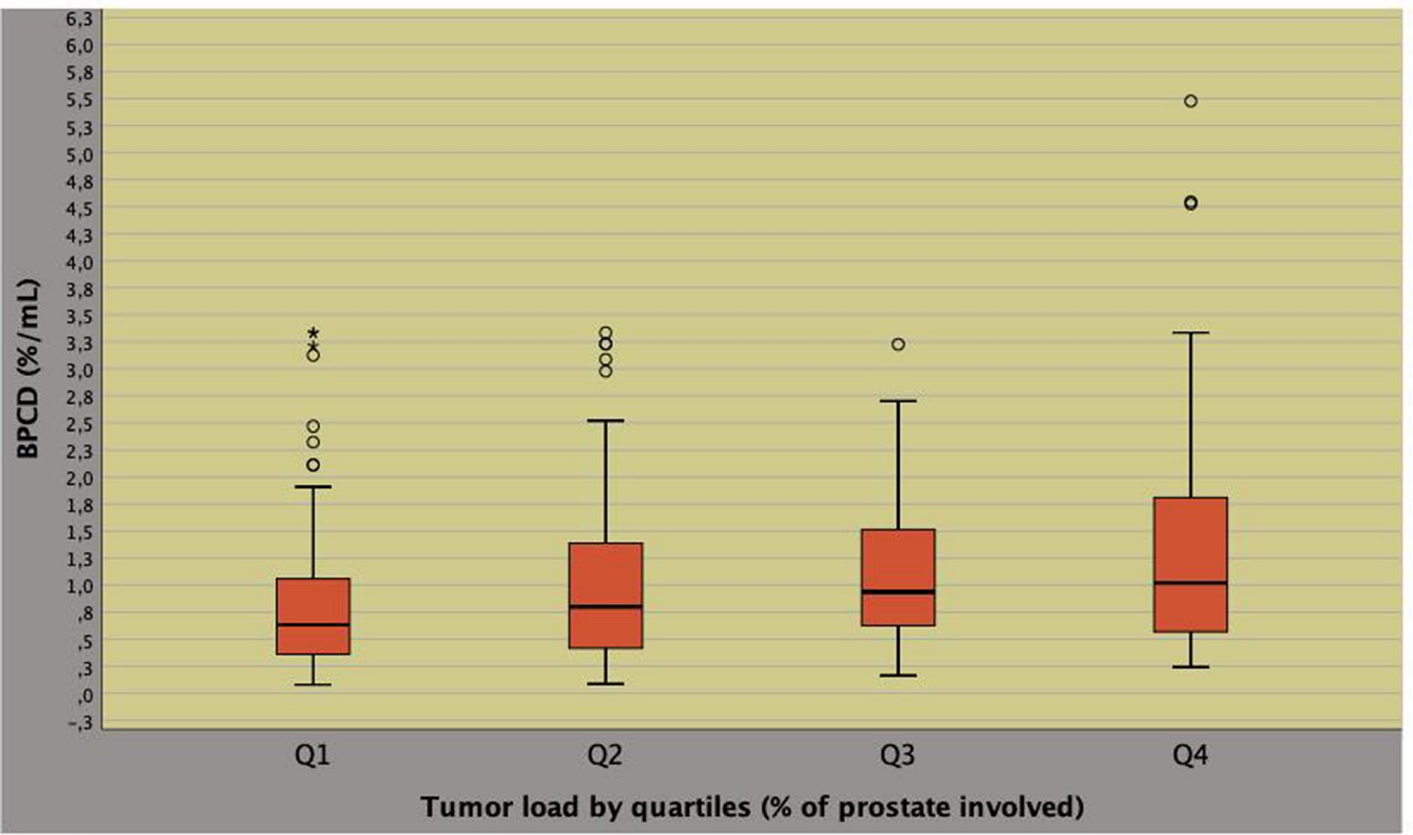
Positive correlation between biopsy tumor load as density of percentage biopsy positive cores (BPCD) and tumor load at surgical pathology (Pearson’s correlation coefficient, *r* = 0.247; *p* < 0.0001)

As shown in Fig. 1, TL correlated to the pathology ISUP system (Pearson’s correlation coefficient, *r* = 0.227; *p* < 0.0001) and to BPCD (*r* = 0.247; *p* < 0.0001), as illustrated in Fig. 2. As shown in Table 2, BPCD and TL both correlated to either ETD or PSAD according to a positive pattern for the former (*r* = 0.430; *p* < 0.0001) as for the latter (*r* = 0.393; *p* < 0.0001). Furthermore, BPCD also correlated to physical factors including either age or BMI according to an inverse pattern, as well. Unfavorable disease correlated positively to either BPCD or pathology tumor load.

**Table 2:** Associations of specimen and biopsy tumor load features with clinical and pathological factors in 430 prostate cancer patients classified as intermediate risk class

Statistics	Tumor load at pathology (TL)		Tumor load density at biopsy (BPCD)	
	*r*	*p* value	*r*	*p* value
Clinical factors				
Age	**− **0.008	0.864	**− 0.115**	**0.017**
BMI	**− **0.075	0.121	**− 0.136**	**0.005**
ASA	0.037	0.448	**− **0.032	0.509
ET	0.042	0.380	0.062	0.199
PSA	**0.137**	**0.004**	**− **0.066	0.175
PV	**− 0.143**	**0.003**	**− 0.464**	**< 0.0001**
BPC	**0.212**	**< 0.0001**	**0.808**	**< 0.0001**
ISUP	0.018	0.708	**− **0.007	0.880
cT	0.040	0.405	0.082	0.090
PSAD	**0.224**	**< 0.0001**	**0.393**	**< 0.0001**
ETD	**0.097**	**0.044**	**0.430**	**< 0.0001**
Pathological factors				
PW	**− 0.135**	**0.005**	**− 0.323**	**< 0.0001**
ISUP	**0.227**	**< 0.0001**	0.057	0.236
pT	0.228	**< 0.0001**	**0.095**	**0.049**
SM	0.261	**< 0.0001**	0.083	0.084
Unfavorable disease				
Upgrading	0.167	**0.001**	**0.102**	**0.034**
Upstaging	0.256	**< 0.0001**	**0.129**	**0.008**
Upgrading and/or upstaging	0.242	**< 0.0001**	**0.114**	**0.018**

*r*, Pearson's correlation coefficient; see also Table 1

**Fig. 3:**
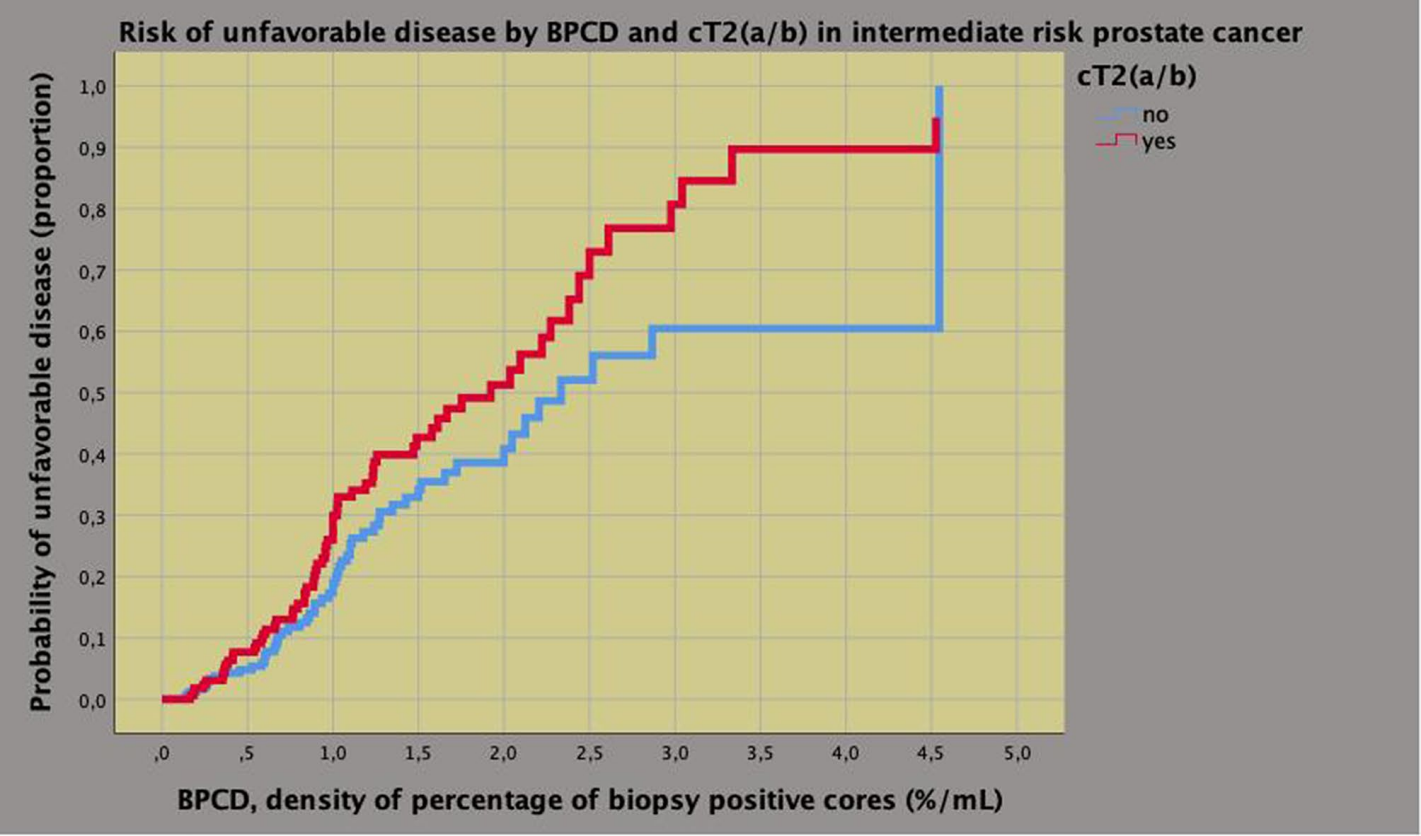
Bivariate clinical model predicting unfavorable disease by percentage of biopsy positive cores (BPCD) and cT stage (cT2a/b versus cT1c) in intermediate risk prostate cancer. The model shows an estimated probability of 0.20 for BPCD = 1.0 (%/mL), which ranks up to 0.30 for clinical stage cT2 (a/b); see manuscript for further details

Associations of UD with clinical and pathological features is reported in supplementary Table 1. On multivariate analysis, the risk of UD was increased by BPCD (odds ratio, OR = 1.319; 95% CI 1.014–1.716; *p* = 0.039) and cT (OR = 2.112; 95% CI 1.375–3.243; *p* = 0.001) for the clinical model as well as by TL (OR = 1.031; 95% CI 1.015–1.047; *p* < 0.0001) and by R1 (OR = 2.250; 1.393–3.634; *p* = 0.001) for the pathological model; furthermore, specificity, sensitivity and overall accuracy resulted, respectively, 99.4%, 3.3% and 72.1% for the former as well as 94.2%, 13.9% and 71.4% for the latter. The bivariate clinical model is depicted in Fig. 3 that shows a significant risk of UD at BPCD = 1.0 (%/mL) that is 0.2 for cT1c stage as well as 0.3 for cT2 (a/b) disease; further details are illustrated in the diagram.

### ETD associations

**Fig. 4:**
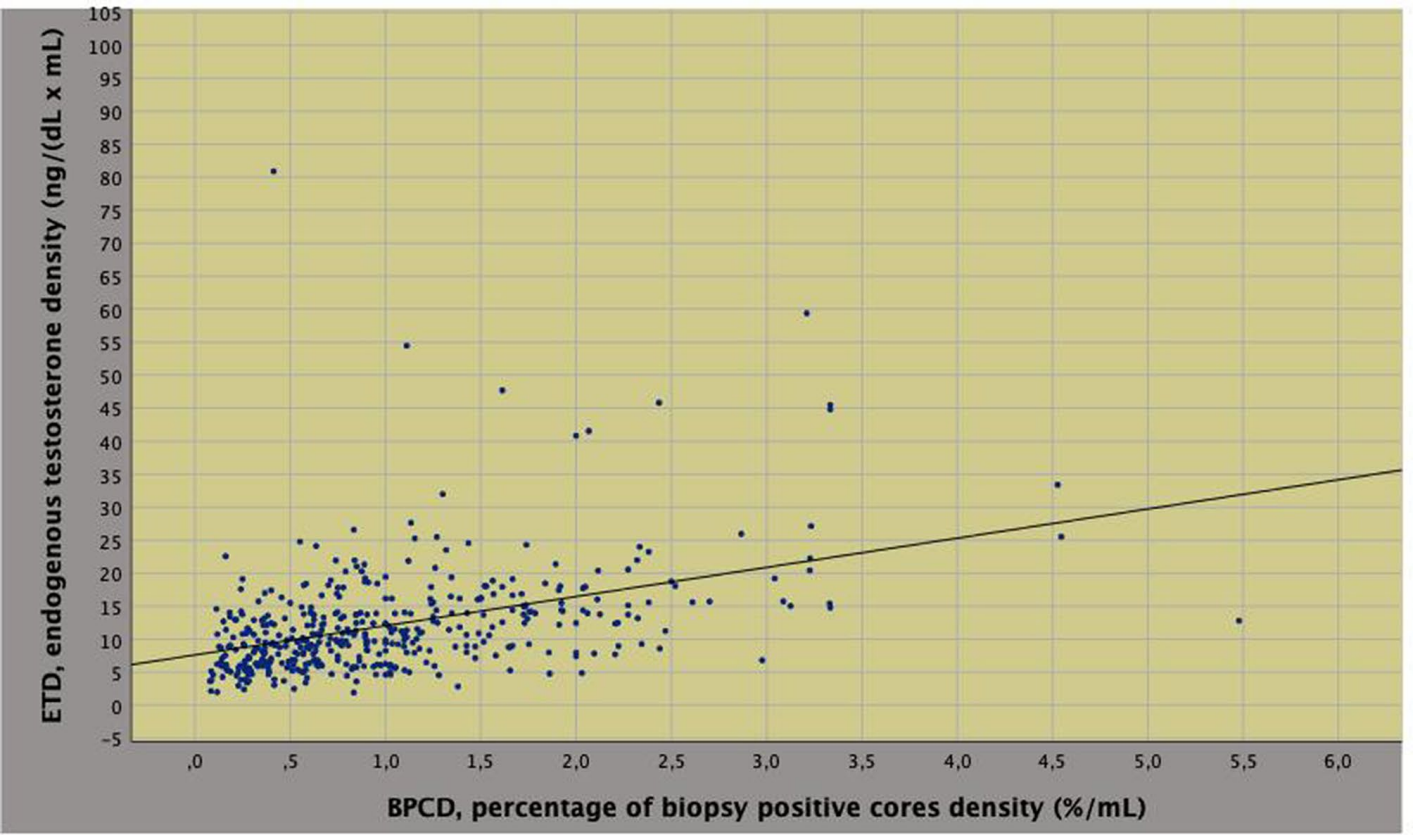
Positive correlation between density of percentage of biopsy positive cores (BPCD) and endogenous testosterone density (ETD) in intermediate risk prostate cancer

**Fig. 5:**
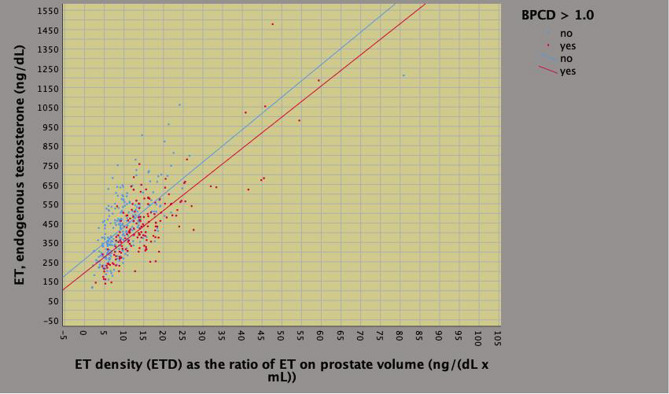
Positive correlation between endogenous testosterone density (ETD) and endogenous testosterone ET in intermediate risk prostate cancer

On univariate analysis, biopsy tumor load density was predicted by endogenous physical factors including age, BMI, ETD, PSAD as well as by pathological features including ISUP grade, pT and specimen tumor load, as well. Among predictors, ETD correlated to either BMI or PSAD according to a pattern that was inverse for the former (*r* = 0.393; *p* < 0.0001) and direct for the latter (*r* = 0.393; *p* < 0.0001). On multivariate analysis, BPCD was independently predicted by PSAD (regression coefficient, *b* = 1.549; 95% CI 0.936–2.162; *p* < 0.0001), ETD (*b* = 0.032; 95% CI 0.023–0.040; *p* < 0.0001) and specimen tumor load (*b* = 0.009; 95% CI 0.005–0.014; *p* < 0.0001), as well. So far, as BPCD increased, ETD and ET levels increased accordingly (Fig. 4); however, patients with higher tumor load densities (BPCD > 1.0%/mL) had significantly lower mean levels of ET (Fig. 5, Table 3); furthermore, as BPCD increased, PSAD and PSA levels increased accordingly, however, larger tumor loads associated with lower PSA levels (Supplementary Fig. 1). The positive correlation between ETD and PSAD is shown in Supplementary Fig. 2 (*r* = 0.393; *p* < 0.0001).

**Table 3:** Associations of clinical and pathological factors with biopsy tumor load density measured as the quotient of percentage of positive cores and prostate volume

Statistics	Univariate analysis		Multivariate analysis		Multivariate model	
	*b* (95% CI)	*p* value	*b* (95% CI)	*p* value	*b* (95% CI)	*p* value
Age	− 0.014 (− 0.025; − 0.002)	0.017	− 0.007 (− 0.017; 0.003)	0.155		
BMI	− 0.033 (− 0.056; − 0.010)	0.005	− 0.009 (− 0.029; 0.012)	0.404		
PSAD	2.664 (2.072; 3.257)	< 0.0001	1.484 (0.863; 2.105)	< 0.0001	1.549 (0.936; 2.162)	< 0.0001
ETD	0.042 (0.034; 0.050)	< 0.0001	0.031 (0.022; 0.040)	< 0.0001	0.032 (0.023; 0.040)	< 0.0001
TL	0.014 (0.008; 0.019)	< 0.0001	0.008 (0.003; 0.014)	0.003	0.009 (0.005; 0.014)	< 0.0001
pT2	Ref		Ref			
pT3	0.247 (0.066; 0.429)	0.008	0.138 (− 0.024; 0.300)	0.096		

*b* linear regression coefficient, *CI* confidence interval, see also Table 1

## Discussion

Tumor misclassification for grading and staging at diagnosis of PCa is a pivotal issue when managing localized disease. Epstein et al. while investigating on tumor grade correlations at needle biopsy and RP specimens in 7643 patients found out that 36.3% of cases were upgraded from Gleason score 5–6 at biopsy to higher grades at surgical pathology; furthermore, the investigators found out that subjects were more likely to be upgraded for smaller prostates and larger amounts of cancer at biopsy. However, the authors were concerned for these results, which appeared controversial for larger amounts cancer at biopsy should decrease such risk. The researchers concluded that further studies should address such features, as well [12]. Actually, the new tumor grading system is more accurate than the Gleason score system in predicting prognosis after RP. Indeed, *Epstein and associates* while investigating in 20,845 subjects treated with RP and having a mean follow-up of 3 years, showed that 5-year biochemical risk free survival for the five ISUP categories at surgical pathology were 96%, 88%, 63%, 48% and 26%, respectively; so far, tumor upgrading for clinical misclassification of the primary tumor is a critical issue for drawbacks on prognosis [13]. In patients undergoing radiation treatment, Zumsteg et al. have demonstrated that intermediate risk PCa is a heterogenous category for including favorable and unfavorable prognostic risk groups with the latter expressing more aggressive features (primary Gleason pattern 4, BPC at least 50% or cT2b-c, PSA 10–20 or Gleason score 7) when compared with the former. As a result, the latter associated with an increased risk of PCa specific mortality; however, the study was limited for being retrospective, for length of follow-up (71 months), for androgen deprivation therapy not being a randomized variable, for not evaluating PSAD and percentage of cancer involving biopsy cores [14].

Overall upgrading/upstaging rates of IR patients at surgical pathology are difficult to assess in contemporary cohorts because of inclusion criteria, which are not the same for the two main systems referring to the National Comprehensive Cancer Network (NCCN) and the European Association of Urology (EAU) for the latter not including clinical stage T2c, which is instead considered by the former [2, 15]. Recently, Stolzenbach et al. investigated on an operated cohort including of 4,048 patients during a time period between 2000 and 2018 in a large European tertiary referral center and found out that overall tumor upgrading occurred in 2.1% of cases while tumor upstaging in 31.4% of subjects. They further stratified the patient population in favorable and unfavorable subsets, and demonstrated that upgrading and upstaging rates were significantly higher for the latter (3.8% and 30.6%, respectively) compared with the former (0.5% and 20.4%, respectively). Moreover, unfavorable patients were more likely to be older with smaller prostates but higher PSA levels and larger biopsy tumor loads. The study was biased for being retrospective, for including cT2c cases, for not being reviewed by central pathology, for missing evaluation of PSAD mpMRI parameters, as well [16]. Su et al. investigated on 4322 IR patients and found out that adverse surgical pathology, which included ISUP > 2, seminal vesicle invasion and pN1 disease, occurred in 34.3% of subjects; specifically, ISUP > 3 occurred in 7.4% of cases, pT3b in 6.6% of subjects, and pN1 in 2.2% of cases; however, this study had several limitations for being retrospective, for incomplete biopsy data, for missing evaluations of mpMRI and genetic tests, for including cT2c cases; furthermore, pT3a disease and surgical margins were not evaluated as adverse pathology, as well [4]. Ploussard et al*.* investigated on 2323 IR patients with Gleason pattern 3 + 4 and assessed that the overall rate of unfavorable disease (upgrading and/or upstaging) was 46%. The authors showed that patients with unfavorable surgical pathology were more likely to have higher PSA and PSAD values, cT > 1 and a number of positive cores > 2; however, the study was biased by several limitations including the retrospective and multicenter nature, absence of central pathology review and missed evaluation of maximal percent of cancer involvement or total tumor length; furthermore, the study included patients having PSA values > 10 ng/mL, cT2c, and BPC > 50%, as well [17]. Yang et al. investigated on 10,089 favorable intermediate risk patients (Gleason pattern 3 + 4, PSA < 10 ng/mL, cT 1c-2a) and found out that adverse surgical pathology (tumor upgrading and/or upstaging) occurred in 30.3%. Specifically, Gleason score > 7 was detected in 3.9% of cases and adverse staging (pT3a-3b and /or pN1 disease) in 17.1%; Patients more likely to have adverse surgical pathology were older, had higher PSA levels, higher clinical stage (cT2a vs T1c) and higher percentages of biopsy positive cores. However, the study was limited by several factors including the retrospective nature, absence of mpMRI findings; importantly, percentage of PCa involving each core, PSAD, percentage of Gleason pattern 4 and perineural invasion were not evaluated, as well [5].

Our study confirmed that IR disease is a heterogenous group of patients who may occult aggressive disease at surgical pathology including upgrading and/or upstaging at a rate of 28%, which means that almost one out of three cases. Patients presenting with higher BPC densities and cT2a/b were more likely to have unfavorable disease according to the risk model; as reported, specificity was 99.35%, which represents the probability that the patients will be identified as not occulting unfavorable disease at surgical pathology (the higher the numerical value of specificity, the less likely the model returns false negative results); moreover, although specificity was low, overall accuracy was 72.1%; finally, we identified BPCD at 1.0%/mL as a critical point for discriminating subgroups. Our study has shown that BPC, ET and PSA should be adjusted for relative prostate volumes in order to relate dynamic changes occurring in the system connecting BMI, PV, tumor load and gonadal axis, as well. As tumor load density increased at biopsy, ETD and PSAD increased accordingly; however, mean levels of either ET or PSA were significantly lower for patients occulting unfavorable disease; furthermore, we identified as a cut point at BPCD = 1.0 (%/mL) for stratifying risk groups (see also Figs. 4 and 5). Overall, these results, which represent a novelty for literature dealing with this subject, have implications in interpreting PCa biology as well as in managing IR cases.

The subject dealing with associations of ET with unfavorable PCa at surgical pathology is controversial for controlled studies are missing; moreover, ET should be measured periodically, as suggested [18, 19]. We do not go into the details of all these studies that show three main outcomes over and over again for ET may associate or not with the risk of unfavorable disease at surgical pathology; when associations have been detected, either patients with lower or higher ET levels may have an increased risk of occulting unfavorable disease, which can include tumor upgrading and/or upstaging; however, none of these studies have investigated specifically in in the IR group, as well [18, 19]. Although adjusting ET for prostate volumes is a novelty for IR, we have investigated this feature in the low-risk category; specifically, we showed that ETD associated with the risk of high tumor load (percentage of cancer involving at least 20% of the gland) that predicted the risk of unfavorable disease at surgical pathology; furthermore, although that study is not comparable with the present one for risk class category and for not evaluating BPCD, both investigations showed efficacy of ETD for evaluating hormone dynamics in PCa patients, as well [8].

Our study showed that patients presenting with either lower ET or PSA levels, when related to respective densities, were more likely to have unfavorable disease in the surgical specimen. These results can be explained by considering dynamics relating ET, PSA and PV variations, which occur in the aging male, as well [6]. Age and BMI are the main physical factors impacting on hormonal dynamics with the former associating with lower free testosterone levels and the latter with either lower free or total testosterone levels, as well [20]. Literature evidences have shown that prostate growth is strictly dependent on ET at very low levels; however, when ET levels decrease down to critical points, testosterone deficiency has drawbacks on differentiation and division of androgen dependent cells; furthermore, as prostate cells are continuously exposed to low ET levels, the risk of cancer induction increase, accordingly [21, 22]. The results of our study support these findings; furthermore, in another large investigation, we have also shown that as severity of prostate growing disorders worsen, ET levels decrease, accordingly. So far, in the aging male, ET levels correlate to changing patterns involving BMI, gonadal axis and prostate growing disorders; so far, as factors defining the system worsen, the risk of disease progression increases, accordingly [23–26].

Our study has implications in managing IR patients occulting unfavorable disease. Beyond standard factors, the risk of tumor upgrading and/or upstaging can be also evaluated by BPCD at a critical point of 1.0%/mL corresponding to a probability of at least 0.20. Moreover, subjects with lower mean levels of either ET or PSA, when related to respective densities, are more likely to have unfavorable disease in the surgical specimen.

Our study has limitations. The study was retrospective and prostate volumes were not all measured in our institution. ET was measured during preoperative evaluation only.

Considering the entire cohort, around 30% of biopsies were performed in different institutions and in most cases the biopsy report was not available. Therefore, it was not possible to investigate differences in tumor upgrading and upstaging after surgery between internal and outside patients, as well as between trans-rectal and trans-perineal approach. However, not difference have been previously found on PCa detection rate between these two approaches during our internal analysis [27], and the results were confirmed by a recent meta-analysis [28]. Moreover, we have no found differences between biopsies performed in our institution and those performed elsewhere considering upgrading rates from low to intermediated- and or high-risk disease [29].

Additionally, a central pathology review of biopsies performed elsewhere was not performed. Results of mpMRI were not evaluated for not being available in all patients. Analysis of maximal cancer involvement of each core.

However, all prostate specimens were assessed by our dedicated pathologist. ET was measured in the morning, which is the appropriate interval for evaluating the levels of the hormone, which decrease in the afternoon, as well [30]. Data collection were prospectively computed. It was single-center study and the patient population was homogenous for ethnicity (Caucasian) and for ET measurements, which were all performed at our laboratory.

## Conclusions

As ETD increased, tumor load density and tumor load at surgical pathology increased. Furthermore, patients with lower ET levels were more likely to occult unfavorable disease at surgical pathology. In IR disease, further studies are required to address the issue of the influence of tumor load and unfavorable disease on ET levels and relative densities.

## Supplementary Information

Below is the link to the electronic supplementary material.

Supplementary file1 (XLSX 11 KB)

Supplementary Fig. 1. Positive correlation between prostate specific antigen density (PSAD) and PSA in intermediate risk prostate cancer (JPG 46 KB)

Supplementary Fig. 2 Positive correlation between endogenous density (ETD) and prostate specific antigen density (PSAD) in intermediate risk prostate cancer (Pearson’s correlation coefficient, r = 0.393; p < 0.0001). (JPG 52 KB)
