# Supplementary figures and images for: Endogenous testosterone density predicts unfavorable disease at final pathology in intermediate risk prostate cancer
Source: Int Urol Nephrol. 2021 Sep 27;53(12):2517–26. doi: 10.1007/s11255-021-02990-9 (PMC8599400; doi:10.1007/s11255-021-02990-9)

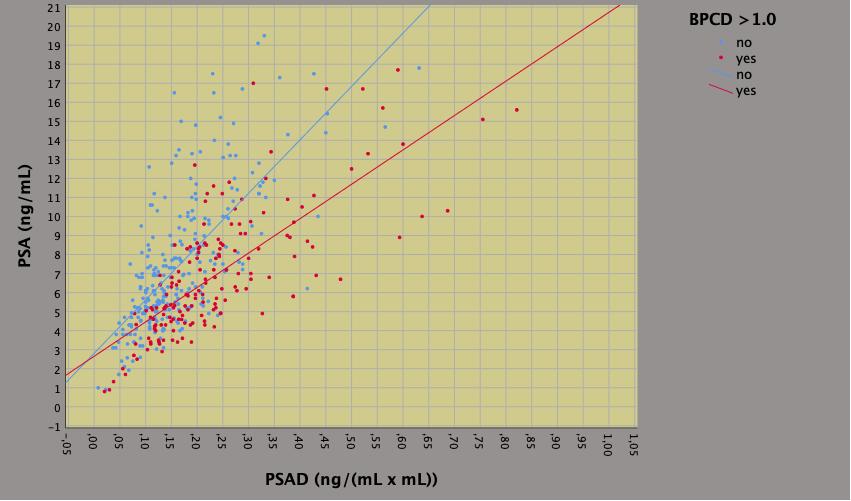

Supplement: Supplementary file 2 — Supplementary Fig. 1. Positive correlation between prostate specific antigen density (PSAD) and PSA in intermediate risk prostate cancer (JPG 46 KB) [file 11255_2021_2990_MOESM2_ESM.jpg]

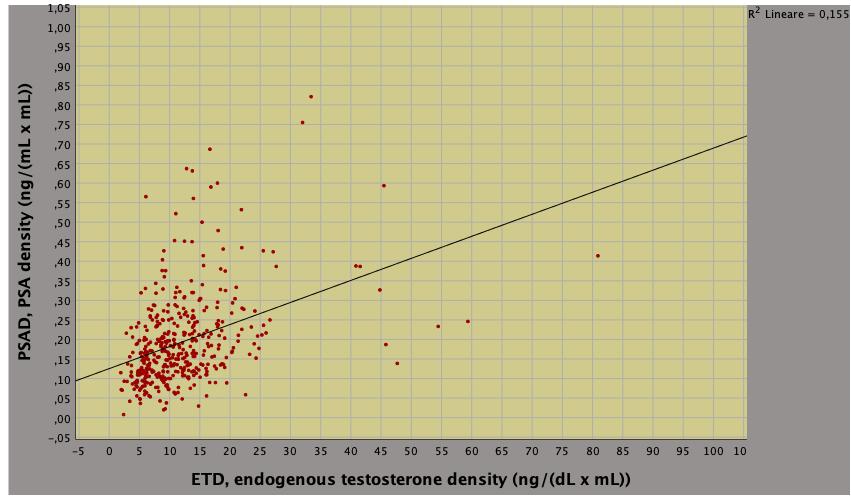

Supplement: Supplementary file 3 — Supplementary Fig. 2 Positive correlation between endogenous density (ETD) and prostate specific antigen density (PSAD) in intermediate risk prostate cancer (Pearson’s correlation coefficient, r = 0.393; p < 0.0001). (JPG 52 KB) [file 11255_2021_2990_MOESM3_ESM.jpg]
